# Supplementary material for: Clinical diagnosis of TIA or minor stroke and prognosis in patients with neurological symptoms: A rapid access clinic cohort
Source: PLoS One. 2019 Mar 19;14(3):e0210452. doi: 10.1371/journal.pone.0210452 (PMC6424476; doi:10.1371/journal.pone.0210452)
Supplement: S1 Table — (DOCX) [file pone.0210452.s001.docx]

**S1 Table**

Unadjusted and adjusted hazard ratios for ischaemic stroke or MI during follow up in patients with a diagnosis of definite or probable stroke or TIA versus patients with another diagnosis in period 0–90 days and 90–3000 days, for different definitions of recurrent stroke: a ischaemic stroke >7 days) (primary analysis); 0 days; and >30 days after the qualifying event.

|  |  | **Hazard ratio (95% CI)** | | |  |
| --- | --- | --- | --- | --- | --- |
|  | **>7 days** | | **0 days** | **>30 days** | |
|  |  | |  |  | |
| **0-90 days** |  | |  |  | |
| **Unadjusted** | 2.88 (2.19-3.80) | | 3.39 (2.81-4.08) | 3.33 (2.10-5.26) | |
| **Adjusted^1^** | 2.83 (2.13-3.76) | | 3.37 (2.79-4.08) | 3.15 (1.97-5.03) | |
| **Adjusted^2^** | 2.76 (2.08-3.68) | | 3.36 (2.78-4.07) | 3.05 (1.90-4.88) | |
|  |  | |  |  | |
| **91-3000 days** |  | |  |  | |
| **Unadjusted** | 1.81 (1.49-2.20) | | 1.79 (1.47-2.18) | 1.79 (1.48-2.16) | |
| **Adjusted^1^** | 1.52 (1.25-1.86) | | 1.49 (1.22-1.83) | 1.51 (1.25-1.84) | |
| **Adjusted^2^** | 1.40 (1.14-1.70) | | 1.36 (1.11-1.67) | 1.39 (1.14-1.68) | |

1. Adjusted for age and sex
2. Adjusted for age, sex, MI, previous stroke, transient ischaemic attack, atrial fibrillation, angina, cardiac failure, peripheral vascular disease, diabetes
