# Supplementary material for: Clinical diagnosis of TIA or minor stroke and prognosis in patients with neurological symptoms: A rapid access clinic cohort
Source: PLoS One. 2019 Mar 19;14(3):e0210452. doi: 10.1371/journal.pone.0210452 (PMC6424476; doi:10.1371/journal.pone.0210452)

**S1 Figure** Interactions between clinical characteristics and hazards of stroke or MI 90 days onwards


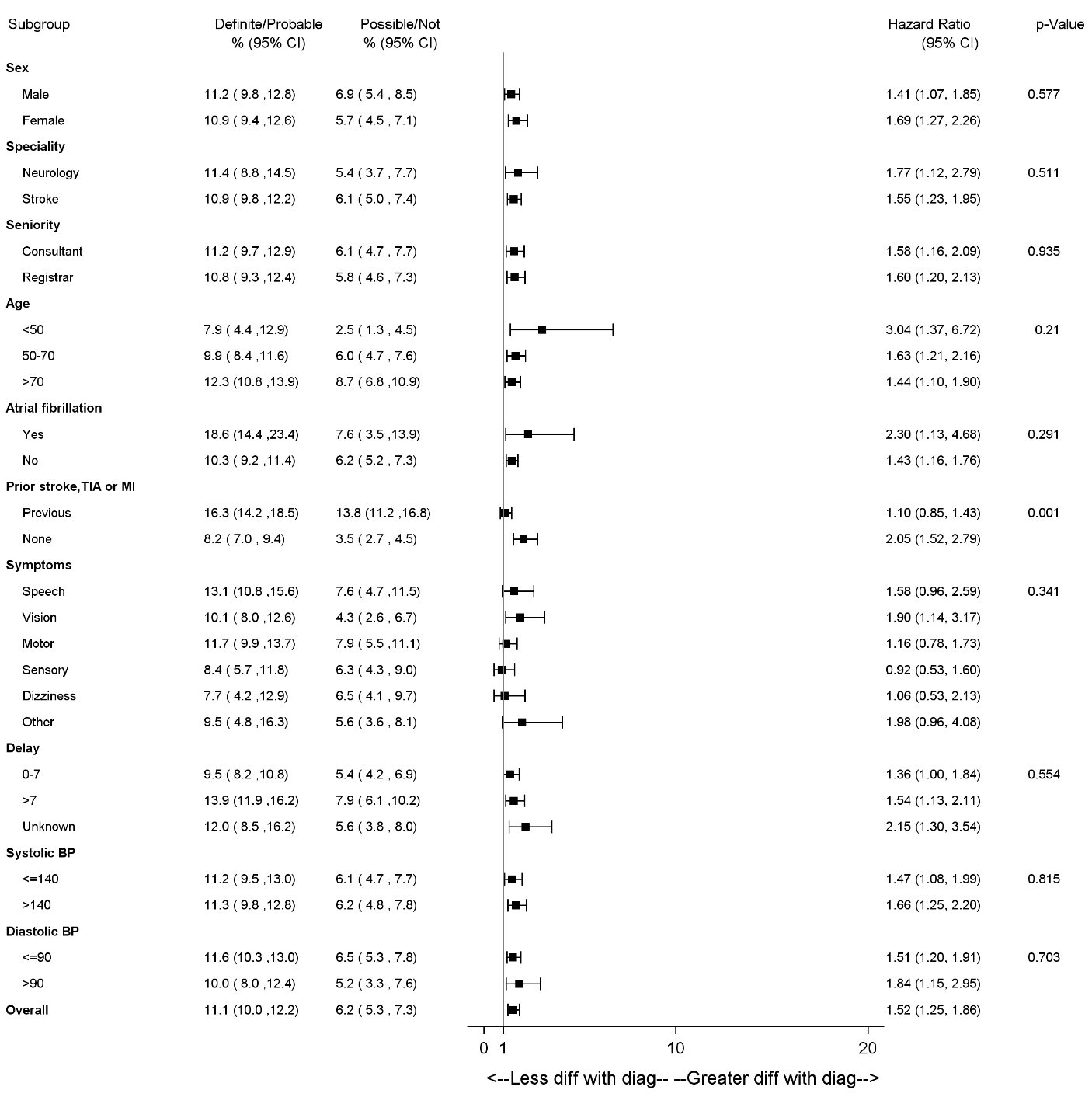

Supplement: S1 Fig — (DOCX) [file pone.0210452.s002.docx]
