# Supplementary material for: Clinical diagnosis of TIA or minor stroke and prognosis in patients with neurological symptoms: A rapid access clinic cohort
Source: PLoS One. 2019 Mar 19;14(3):e0210452. doi: 10.1371/journal.pone.0210452 (PMC6424476; doi:10.1371/journal.pone.0210452)

**S2 Figure:** Cumulative incidence curves of ischaemic stroke or myocardial infarction in follow up in patients with a diagnosis of definite, probable, possible stroke or TIA and non-cerebrovascular diagnosis, with 95% CI.


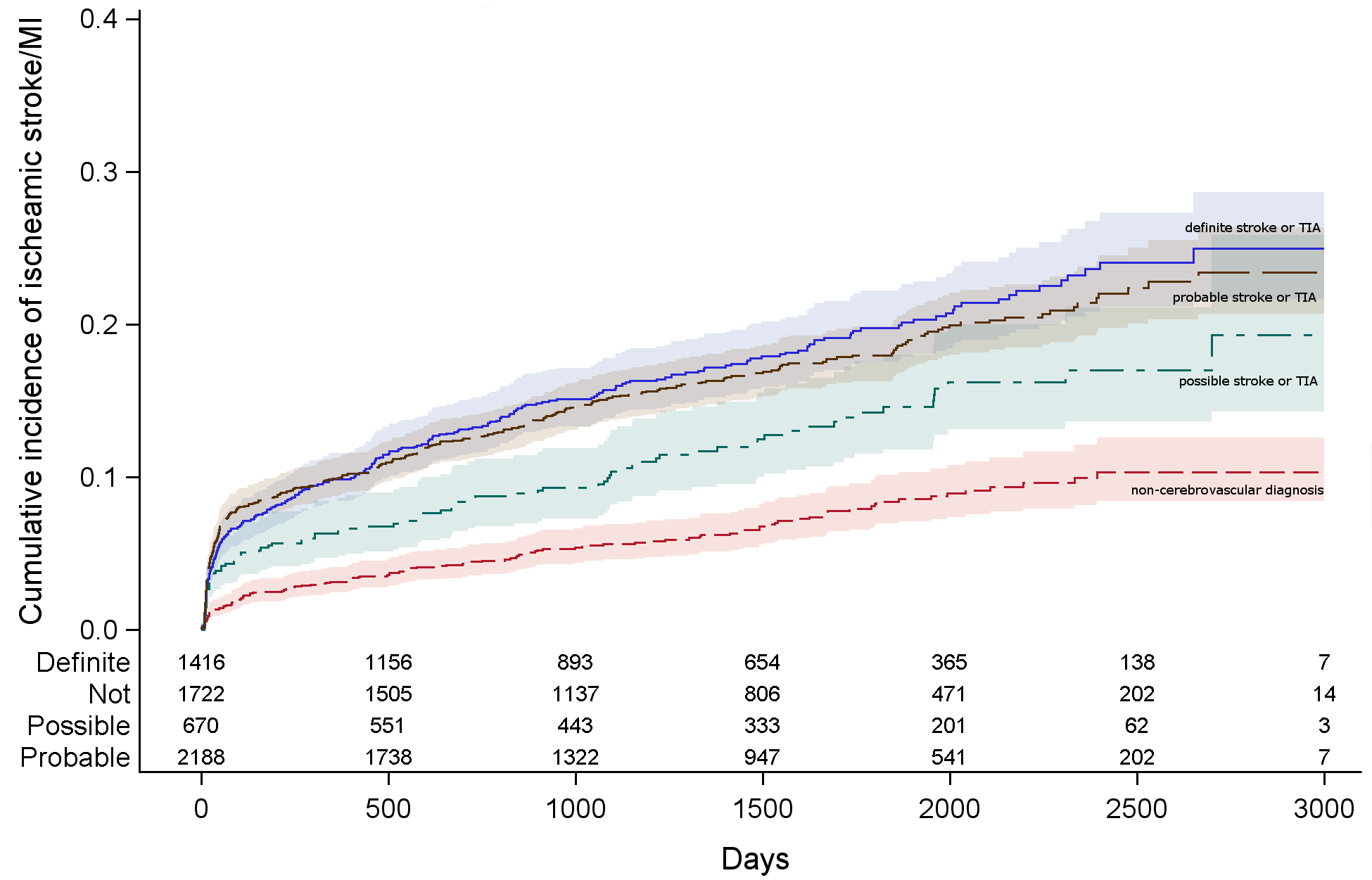

Supplement: S2 Fig — (DOCX) [file pone.0210452.s003.docx]
